# Supplementary material for: Regulations on the Use of Antibiotics in Livestock Production in South America: A Comparative Literature Analysis
Source: Antibiotics (Basel). 2023 Aug 9;12(8):1303. doi: 10.3390/antibiotics12081303 (PMC10451520; doi:10.3390/antibiotics12081303)
Supplement: Supplementary file 1 [file antibiotics-12-01303-s001.zip › antibiotics-2501335-supplementary.pdf]

Table S1: Legislation, Guidelines, and other documents on Veterinary Antibiotic Use in South American Countries

**Argentina**	
**Year**	**Legislation/Other Documents**
2009	Resolución n° 63
2010	Resolución 542- Establécense requisitos sobre instalaciones, bioseguridad, higiene y manejo sanitario, para el registro y habilitación sanitaria de establecimientos avícolas de producción
2011	Resolución n°666- Créase el Libro de Registros de Tratamientos, en el ámbito de los establecimientos pecuarios de producción de animales para consumo humano en todo el Territorio Nacional, con el objeto de registrar todos y cada uno de los tratamientos vinculados a la administración de productos veterinarios sobre los animales existentes en los mismos
	Resolución n°323- Procedimiento de notificación de eventos. Establécese el Procedimiento para la notificación de eventos relacionados con la utilización de Productos Veterinarios aprobados.
2015	Resolución n° 594- Se aprueba la norma técnica de alimentos para animales de lá República Argentina
	Resolución n° 591- Crea el Programa Nacional de Vigilancia de la Resistencia a Los antimicrobianos em animales destinados al consumo humano
2018	Resolucíon 575- Establece requisitos para el bienestar animal de los pollos de engorde
	Resolución n° 1119- Adecua la citada Resolución Nº 594/15 con relación a las sustancias evaluadas
2019	Resolución n° 1642- MARCO REGULATORIO PRODUCTOS VETERINARIOS
	Resolución n°1642-Anexo III Formulario de Inscripción para produtos farmacológicos de uso veterinário
	Resolución n° 22- Productos veterinarios: prohibición de elaboración, distribución, importación, uso y tenencia
	Ley 14.072- Del Ejerciio de la Medicina Veterinária
	Resolución 1697- Exigencias mínimas relativas al bienestar animal
-	Manual de Bienestar Animal: En Plantas de Aves y Lagomorfos
-	Manual de bienestaranimal:un enfoque práctico para el buen manejo de especies domésticas durante su tenencia, producción, concentración, transporte y faena
-	Controle de Resíduos (PLAN CREHA)
**Brazil**	
**Year**	**Legislation/Other Documents**
1996	Portaria 74- Roteiro para registro de produtos biológicos de uso veterinário
2003	Instrução Normativa n° 9- Proibir a fabricação, a manipulação, o fracionamento, a comercialização, a importação e ouso de princípios ativos de cloranfenicol.
2004	Decreto n°5053- Regulamento de Fiscalização de Produtos de Uso Veterinário e dos Estabelecimentos que os Fabriquem ou Comerciem, e dá outras providências
2005	Instrução Normativa n° 65- Regulamento técnico sobre fabricação e o emprego de alimentos para animais contendo medicamentos
2009	Instrução Normativa n°22- Regulamenta a embalagem, rotulagem e propaganda dos produtos destinados à alimentação animal
	Instrução Normativa n°26- Proibir a fabricação, a manipulação, o fracionamento, a comercialização, a importação e ouso de princípios ativos de cloranfenicol.
2012	Instrução Normativa n° 14- Proibi a utilização de espiramicina e eritromicina como melhoradores de desempenho em animais.
2015	Decreto n°8448- Altera o regulamento de fiscalização de produtos de uso veterinário e dos estabelecimentos que os fabriquem ou comerciem.
2016	Instrução Normativa n° 45- Proibi a utilização de sulfato de colistina como melhoradores de desempenho em animais
	Resolução 1138- Conselho Federal de Medicina Veterinária
2018	Instrução Normativa n°54- Aprova o regulamento técnico para o registro de aditivos antimicrobianos melhores de desempenho e aditivos anticoccidianos administrados via alimentação animal
2020	Instrução Normativa n°1- Proibi a utilização de tilosina, lincomicina e tiamulina como melhoradores de desempenho em animais.
	Instrução Normativa 113- Estabelecer as boas práticas de manejo e bem-estar animal nas granjas de suínos de criação comercial
-	Suinocultura: Uma Saúde e um Bem-estar
-	O uso prudente e eficaz de antibióticos na suinocultura
-	Painel de Negócios Inteligente do Ministério da Agricultura e Pecuária e Abastecimento
-	Agromonitora
-	Programa de Vigilância e Monitoramento da Resistência aos antimicrobianos no âmbito da agropecuária
-	Plano Nacional de Controle de Resíduos e contaminantes PNCRC/ANIMAL
**Chile**	
**Year**	**Legislation/Other Documents**
2005	Decreto n°25- Reglamento de productos farmacéuticos de uso exclusivamente veterinario

2010	Resolución n°667- Establece, requisitos para la fabricación, importación, expendio y prescripción de medicamentos veterinários cuya condición de venta sea bajo receta retenida com control de saldo y deroga resolución n° 206 de 2004
2011	Decreto n° 24- Modifica Decreto n° 25 de 2005
2017	Decreto n° 4- Reglamento de alimentos para animales
	Resolución n°6801- Establece requisitos para el registro, comercialización y uso de antimicrobianos
2018	Resolución 4579- Modicase la Resolución Exenta Nº 6.801
2020	Resolución n° 2517- Modifica Resolución nº 6.801 exenta, de 2017
2021	Código de ética profesional
-	Antimicrobianos
-	Manual de Buenas Practicas de Sobre Bienestar Animal em Sistema de Producción Industrial de Cerdos
-	Programa de Control de Residuos em Productos Pecuarios
**Colombia**	
**Year**	**Legislation/Other Documents**
1981	Resolución n° 1326- Se adoptan disposicionae para utilización y comercialización de produtos antimicrobianos de uso veterinario
1984	Resolucion n° 1966-Reglamenta el uso de products o sustancias antimicrobianas como promotores de crecimientos o mejoradores de la eficiencia alimenticia
1996	Resolución n°1056-Dictan disposiciones sobre el control técnico de los Insumos Pecuarios y se derogan las Resoluciones No. 710 de 1981, 2218 de 1980 y 444 de 1993
2000	Ley 576- Expide el código de Etica para el ejercicio professional de la medicina veterinária, la medicina veterinária y zootecnia
2003	Resolución n° 3826 - Establece el cumplimento de las Buenas Prácticas de Manufactura para las empresas productoras por contrato de medicamentos veterinarios
2007	Resolución n° 2341- Reglamentan las condiciones sanitárias y de inocuidad em la producción primaria de ganado bovino y bufalino destinado al sacrifício para consumo humano
	Resolución 2640- Reglamentan las condiciones sanitarias y de inocuidad en la producción primaria de ganado porcino destinado al sacrificio para consumo humano
2008	Resolución n° 3585- Se establece el sistema de inspección, evaluación y certificación oficial de la proucción primaria de leche
2013	Resolución 1382- Establecen los limites máximos para resíduos de medicamentos veterinários em los alimentos de origen animal destinados al consumo humano
2017	Decreto 2113- Se adiciona um capítulo al decreto 1071 de 2015
	Resolución 10204- Se estabelece el Sistema Nacional de Farmacovigilancia para medicamento y biológicos de uso veterinario
2020	Resolución 62542- Se establecen los requisitor y el procedimento para el registro de los medicamentos de uso veterinária ante el ICA
	Resolución n°61252- Establecen los requisitor y el procedimento para el registro de los fabricantes e importadores de alimentos para animales, así como los requisitor y el procedimento para el registro de alimentos para animales y se dictan otras disposiciones
2021	Resolución n°90382-Por medio de la cual se establecen los requisitos para la comercialización, distribución, almacenamiento de los insumos agropecuarios y semillas para siembra.
-	Buenas prácticas em el uso de los medicamentos veterinários y la inocuidade de los alimentos
-	Investigación y Vigilancia Integrada de la Resistencia Antimicrobiana
**Uruguay**	
**Year**	**Legislation/Other Documents**
1993	Resolución n° 328- Control de los alimentos destinados a la nutrición animal a efectos de verificar su composición, calidad y destino
1997	Decreto n°160- Aprobacion del marco regulatorio sobre productos veterinarios en el mercosur
2003	Resolución n° 360- Programa nacional de residuos biologicos en alimentos de origen animal
2004	Decreto n° 177- Establecer que los establecimientos productores de carne y leche con fines comerciales deberán llevar una planilla de registro de utilización de productos veterinarios
2011	Decreto n° 98- prohibicion del uso de antibioticos en la alimentacion para animales ovinos y bovinos
2012	Resolución n°48- Apruébase la Norma Técnica de Buenas Prácticas de Fabricación
2012	Resolución n°89- Aprobar los requisitos para la habilitación e implementación de Buenas Prácticas de Fabricación en los Estabelecimientos Elaboradores de Alimientos para Animales
2015	Resolución n° 193- Regulación de las condiciones de comercialización y uso de antibióticos y antimicrobianos para animales
2018	Decreto n° 389-Reglamentacion de la ley 19.258 relativa a la creacion del colegio veterinario del uruguay
2022	Resolución n°22- Apruébese los Requisitos de bioseguridad y el Procedimiento para la habitación, ampliación y refrendación de establecimientos avícolas.
	Formulario de inscripción para producto farmacológicos de uso veterinario
-	Buenas práticas de uso de medicamentos veterinários
-	Buenas Prácticas Ganaderas: Guía para la implementación en la produción de ganado vacuno de carne
-	Guía de Buenas Prácticas en Bienestar Animal durante la Cría y Faena de aves de producción de carne

-	Control de Venta de produtos veterinarios
-	Uruguay frente al desafío de los antimicrobianos en salud animal y cadenas productoras de alimentos
**Mercosul**	
**Year**	**Legislation/Other Documents**
1993	Resolução n° 11- Marco Regulatório para Produtos Veterinários
1996	Resolução n° 39- regulamentação complementar do marco regulatório de produtos veterinários

Source: Authors based on Brazil: Ministério da Agricultura Pecuária e Abastecimento, Conselho Federal de Medicina Veterinária; Argentina: Servicio Nacional de Sanidady Calidad Agroalimetnaria; Consejo professional de Medicos Veterinarios; Colombia: Ministério de Agricultura y Desarrollo Rural, Instituto Colombiano Agropecuário (ICA), Consejo Profesional de Medicina Veterinárias e Zootecnica; Chile: Serviço Agricola y Ganadero, and Colégio Médico Veterinário de Chile; Urugay: Ministério da Ganaderia Agricultura y Pesca, and Colégio Veterinário de Uruguay; and Mercado Comum do Sul.
